# High diversity, close genetic relatedness, and favorable living conditions benefit species co-occurrence of gut microbiota in Brandt’s vole

**DOI:** 10.3389/fmicb.2024.1337402

**Published:** 2024-02-07

**Authors:** Chaoyuan Cheng, Guoliang Li, Xifu Yang, Jidong Zhao, Jing Liu, Aihua Zheng, Zhibin Zhang

**Affiliations:** ^1^State Key Laboratory of Integrated Management of Pest Insects and Rodents, Institute of Zoology, Chinese Academy of Sciences, Beijing, China; ^2^Chinese Academy of Sciences Center for Excellence in Biotic Interactions, University of Chinese Academy of Sciences, Beijing, China; ^3^Shaanxi Key Laboratory of Qinling Ecological Security, Shaanxi Institute of Zoology, Xi’an, China; ^4^Ministry of Education Key Laboratory for Ecology of Tropical Islands, Key Laboratory of Tropical Animal and Plant Ecology of Hainan Province, School of Life Sciences, Hainan Normal University, Haikou, Hainan, China

**Keywords:** species co-occurrence, biodiversity, social stress, density dependency, phylogenetic relatedness

## Abstract

**Introduction:**

Revealing factors and mechanisms in determining species co-existence are crucial to community ecology, but studies using gut microbiota data are still lacking.

**Methods:**

Using gut microbiota data of 556 Brandt’s voles from 37 treatments in eight experiments, we examined the relationship of species co-occurrence of gut microbiota in Brandt’s voles (*Lasiopodomys brandtii*) with genetic distance (or genetic relatedness), community diversity, and several environmental variables.

**Results:**

We found that the species co-occurrence index (a larger index indicates a higher co-occurrence probability) of gut microbiota in Brandt’s voles was negatively associated with the genetic distance between paired ASVs and the number of cohabitating voles in the experimental space (a larger number represents more crowding social stress), but positively with Shannon diversity index, grass diets (representing natural foods), and non-physical contact within an experimental space (representing less stress).

**Discussion:**

Our study demonstrated that high diversity, close genetic relatedness, and favorable living conditions would benefit species co-occurrence of gut microbiota in hosts. Our results provide novel insights into factors and mechanisms that shape the community structure and function of gut microbiota and highlight the significance of preserving the biodiversity of gut microbiota.

## Introduction

Species co-existence or co-exclusion is an important factor in shaping the community and functions of various ecosystems, and there are several theories on whether two species can coexist. The competitive exclusion principle (i.e., Gause’s law) refers to the difficulty of two species that are closely related or similar to each other to occupy the same or similar ecological niche and thus cannot coexist for a long time (Hardin, 1960). Similarly, the ecological niche theory suggests that two species cannot co-exist if their niches highly overlap (Letten et al., 2017). However, the environmental filtering theory suggests that species with similar environmental demands (e.g., closely related species) are more likely to co-exist (Sommer et al., 2014; Le Bagousse-Pinguet et al., 2017).

Species co-occurrence has been widely used to test these hypotheses over the past few decades (Letten et al., 2017; Abbate et al., 2018; Wang et al., 2022). The metrics used to calculate species co-occurrence can be classified into matrix-level (Gotelli, 2000; Ulrich et al., 2009) and pairwise (Sfenthourakis et al., 2006; Veech, 2013) approaches. The matrix-level approach calculates a co-occurrence metric as a property of the entire presence-absence matrix, whereas the pairwise approach measures co-occurrence “species by species” (Veech, 2014). Some metrics, including proportional similarity (Schoener, 1970) and correlation coefficients, are used to examine the co-occurrence of pairwise species, whereas the correlation coefficient metric is more commonly used in studies of microbial co-occurrence networks (Aas et al., 2005; Gross et al., 2010; Faust et al., 2012; Kehe et al., 2021). However, the relationship between species co-occurrence and genetic distance, as well as the effects of other environmental variables (e.g., diversity, diets, or shelter quality), has been rarely investigated (but see Wang et al., 2022).

The gut of most animals is inhabited by taxonomically and functionally diverse symbiotic microbial communities that can affect animal health and are influenced by genetics (Weinstein et al., 2021), diet (Yin et al., 2017; Li et al., 2021a), and environmental factors (Liu et al., 2020, 2022; Rocchi et al., 2022). Gut microbiota dysbiosis refers to an imbalance in the microbial community that disrupts the microbiota functions that are essential for maintaining host health (Kriss et al., 2018). Dysbiosis of the gut microbiota can lead to various diseases, such as inflammatory bowel disease, metabolic disorders, and neurological disorders (Sekirov et al., 2010; Gonzalez et al., 2011; Carding et al., 2015) and is often associated with a reduction in overall microbial diversity (Kriss et al., 2018). Moreover, gut microbiota dysbiosis is often associated with a reduction in overall microbial diversity (Kriss et al., 2018). Studies have shown that unfavorable factors, such as livestock grazing (Li et al., 2019), social stress (Partrick et al., 2018; Liu et al., 2022), and nutritional deficiency (Laitinen and Mokkala, 2019; Zhao et al., 2022), can reduce the gut microbial diversity of animals and negatively affect the physiological functions of hosts. The composition and relationships of gut microbes may be different across hosts (Faith et al., 2013; Risely et al., 2021), and the composition can fluctuate when the host is disturbed by the external environment (Wu et al., 2017; Mardinoglu et al., 2018). When hosts are genetically related, their gut microbial composition and relationships tend to be similar (Roche et al., 2023). In recent years, however, some studies have suggested that the relationships between gut microbes are more universal across hosts (Bashan et al., 2016; Kalyuzhny and Shnerb, 2017; Vila et al., 2020; Roche et al., 2023). However, the impacts of genetic distance (or genetic relatedness) between gut microbes, the diversity of gut microbes of a community, and environmental factors on the co-occurrence of gut microbiota have not been investigated, as far as we know.

The purpose of this study was to examine the relationship of species co-occurrence of gut microbiota in Brandt’s voles (*Lasiopodomys brandtii*) with their genetic distance (or relatedness), community diversity, and several environmental variables (e.g., diet composition, contact type, number of cohabitating voles in the experimental space). We want to test the following hypotheses: (1) The species co-occurrence index should be negatively correlated with the genetic distance between two paired amplicon sequence variants (ASVs) (or genera) of gut microbes in each of the eight experiments, as predicted by the environmental filter hypotheses. Alternatively, the species co-occurrence index should be positively associated with genetic distance, according to the competitive exclusion principle or niche theory. (2) Species co-occurrence index should be positively associated with the diversity of gut microbiota or favorable environments (often positively associated with high diversity).

## Materials and methods

### Study subjects

Brandt’s voles (*Lasiopodomys brandtii*) are social animals with a polygamous mating system (Batsuren et al., 2022). Their population irrupts irregularly every 3–5 years, and both intrinsic (density and social conflict) and external (rainfall, grazing, and predators) factors have been shown to affect their population growth (Zhang et al., 2003; Li et al., 2021b). Moreover, studies have shown that diet, crowding, and photoperiod can significantly alter the composition of gut microbiota in Brandt’s voles (Zhang et al., 2018; Li et al., 2019; Liu et al., 2020; Zhao et al., 2022; Zhu et al., 2022). In this study, all experimental voles were from our laboratory colony maintained at the Institute of Zoology at the Chinese Academy of Sciences or from our field station in Maodeng Pasture, Xilinghaote, Inner Mongolia, China.

### Experiments and samples

The 16S rRNA gene sequence data of the V3-V4 region of fecal gut microbiota of 556 Brandt’s voles (Table 1) were obtained from previously published studies from our research group (Liu et al., 2020, 2022; Li et al., 2021a). The forward primer was 341F (CCTAYGGGRBGCASCAG) and the reverse primer was 806R (GGACTACNNGGGTATCTAAT) in each study. The region and lengths of sequences were identical between each of the studies. The data were compiled from eight experiments (with 37 treatment groups) in three independent studies: Study 1 (Li et al., 2021a), Study 2 (Liu et al., 2020), and Study 3 (Liu et al., 2022; see Table 1). Individuals in each treatment experienced the same external environment (treatment), which provided us with the opportunity to study the effects of genetic distance, community diversity, and other environmental factors on species co-occurrence between paired ASVs of the gut microbiota in Brandt’s voles. For detailed information about experiments and treatments, see Supplementary information.

**TABLE 1:** Summary of the 37 treatment groups from 8 experiments.

Study	Experiment code	Experimental condition	Treatment	Treatment ID	No. voles	Diet	Physical contact
**Study 1**	Experiment 1	Lab	DSR1	E1_F1	1	Grass	No
**Study 1**	Experiment 1	Lab	DSR2	E1_F2	1	Grass	No
**Study 1**	Experiment 1	Lab	DSR3	E1_F3	1	Grass	No
**Study 1**	Experiment 1	Lab	DSR4	E1_F4	1	Grass	No
**Study 1**	Experiment 1	Lab	DSR5	E1_F5	1	Grass	No
**Study 1**	Experiment 1	Lab	DSR6	E1_F6	1	Grass	No
**Study 1**	Experiment 1	Lab	DSR7	E1_F7	1	Grass	No
**Study 1**	Experiment 1	Lab	DSR8	E1_F8	1	Grass	No
**Study 2**	Experiment 2	Lab	HC without physical contact	E2_H	9	Chow	No
**Study 2**	Experiment 2	Lab	LC without physical contact	E2_L	2	Chow	No
**Study 2**	Experiment 2	Lab	MC without physical contact	E2_M	4	Chow	No
**Study 2**	Experiment 3	Lab	HD with physical contact	E3_H	8	Chow	Yes
**Study 2**	Experiment 3	Lab	LD with physical contact	E3_L	2	Chow	Yes
**Study 2**	Experiment 3	Lab	MD with physical contact	E3_M	4	Chow	Yes
**Study 3**	Experiment 4	Lab	HD with physical contact	E4_H	8	Chow	Yes
**Study 3**	Experiment 4	Lab	LD with physical contact	E4_L	2	Chow	Yes
**Study 3**	Experiment 4	Lab	MD with physical contact	E4_M	4	Chow	Yes
**Study 3**	Experiment 5	Lab	HD without physical contact	E5_H	8	Chow	No
**Study 3**	Experiment 5	Lab	MD without physical contact	E5_M	4	Chow	No
**Study 3**	Experiment 6	Lab	HD with physical contact	E6_H	8	Chow	Yes
**Study 3**	Experiment 6	Lab	LD with physical contact	E6_L	2	Chow	Yes
**Study 3**	Experiment 6	Lab	MD with physical contact	E6_M	4	Chow	Yes
**Study 3**	Experiment 7	Lab	HC without physical contact	E7_H	8	Chow	No
**Study 3**	Experiment 7	Lab	LC without physical contact	E7_L	2	Chow	No
**Study 3**	Experiment 7	Lab	MC without physical contact	E7_M	4	Chow	No
**Study 3**	Experiment 8	Enclosure	HD in enclosures	E8_H1	48	Grass	Yes
**Study 3**	Experiment 8	Enclosure	LD in enclosures	E8_L1	48	Grass	Yes
**Study 3**	Experiment 8	Enclosure	MD in enclosures	E8_M1	48	Grass	Yes
**Study 3**	Experiment 8	Enclosure	HD in enclosures	E8_H2	48	Grass	Yes
**Study 3**	Experiment 8	Enclosure	LD in enclosures	E8_L2	12	Grass	Yes
**Study 3**	Experiment 8	Enclosure	MD in enclosures	E8_M2	12	Grass	Yes
**Study 3**	Experiment 8	Enclosure	HD in enclosures	E8_H3	12	Grass	Yes
**Study 3**	Experiment 8	Enclosure	LD in enclosures	E8_L3	12	Grass	Yes
**Study 3**	Experiment 8	Enclosure	MD in enclosures	E8_M3	24	Grass	Yes
**Study 3**	Experiment 8	Enclosure	HD in enclosures	E8_H4	24	Grass	Yes
**Study 3**	Experiment 8	Enclosure	LD in enclosures	E8_L4	24	Grass	Yes
**Study 3**	Experiment 8	Enclosure	MD in enclosures	E8_M4	24	Grass	Yes

See the Supplementary information for detailed information on treatments and experiments. HD, high density (high group size and high space shortage); MD, medium density (medium group size and medium space shortage); LD, low density (small group size and low space shortage); HC, high crowding (high group size with no space shortage); MC, medium crowding (medium group size with no space shortage); LC, low crowding (small group size with no space shortage). DSR1–DSR8 represent the eight diet treatments comprising plant species 1–8, respectively, by adding plant species in order from high to low palatability. No. voles, number of cohabitating voles in the experimental space.

### Sequence data processing

We removed barcodes and primers of each sequence and removed short sequences (length < 390) using VSEARCH-2.21.1 (Rognes et al., 2016). To standardize the sample size and avoid the sequence-depth effect, we randomly selected 50,000 sequences for each fecal sample of voles using VSEARCH-2.21.1. We removed singletons and clustered ASVs at a 100% identity threshold using the unoise2 algorithm implemented in the *cluster_unoise* function, which is integrated in VSEARCH-2.21.1. To reduce potential error sequences and facilitate subsequent analysis (e.g., rare ASVs can lead to too many zeros, which would affect the correlation estimates), we removed the ASVs with abundances lower than 0.01% (5,527 ASVs in total).

### Species mapping and function prediction

We employed the SILVA rRNA database version 138^1^ to annotate the taxonomic information for each ASV. The length of the 16S rRNA sequences (in the V3–V4 region for this study) used for taxonomic identification generally can assign most ASVs only to the genus level. Thus, only taxa of genera and above were used for subsequent analyses to ensure accuracy.

We used PICRUSt2 (Douglas et al., 2020) to predict the function of gut bacteria according to each ASV. PICRUSt2 predictions were conducted based on several gene family databases, including the Kyoto Encyclopedia of Genes and Genomes (KEGG) orthologs (KOs) and Enzyme Commission (EC) numbers.

### Genetic distance

Calculating genetic distances require aligned sequences; thus, we aligned the ASV sequences using Muscle version 5 (Edgar, 2021) and calculated the genetic distance between the aligned ASV sequences using MEGA version 11 (Tamura et al., 2021).

### Co-occurrence index of ASVs

We calculated Spearman’s correlation coefficient between pairwise ASVs using their relative abundance within each treatment group. Spearman’s correlation coefficient was used to indicate the tendency of co-existence or co-exclusion between ASVs; a positive coefficient indicated co-existence, while a higher value indicated a higher probability of co-existence, and vice versa (Matchado et al., 2021). To reduce potential errors caused by too many zero values, we removed the ASV pairs whose number of zero values was greater than 50%.

### Network analysis

The co-occurrence network was built by using the workflow provided by CoNet (Faust and Raes, 2016). CoNet is an application integrated in Cytoscape (Shannon et al., 2003) and can detects significant non-random patterns of co-occurrence in incidence and abundance data. We discarded ASVs with less than 20 non-zero values across all samples (filtering) and divided each entry by the sum of its corresponding column (normalization), thus avoiding the inference of spurious associations due to different sequencing depths (Faust and Raes, 2016). We used ASVs as nodes and Spearman’s coefficients between nodes as the weights of the edges. To assess edge significance, bootstrap method was deployed with 100 sub-samplings. The edges with *p*-values greater than 0.05 were removed. The degree in a network refers to the number of edges connecting a selected node to the remaining nodes (Liu et al., 2021). The parameters (e.g., degree) of the network were calculated using Cytoscape version 3.9.1, and four hub nodes (Liu et al., 2021) were determined based on two criteria: (1) degree > 1% of total volume and (2) betweenness centrality > 0.1.

### Regression analysis

First, we used linear regression methods to detect the relationship between the co-occurrence index and the genetic distance of pairwise ASVs. For analyses (Table 1) that required ASV selection based on *p*-values, we adjusted the *p*-values using the Benjamini–Hochberg method to calculate the false discovery rate (FDR) as follows:

FDR=p×N/Rank

here, FDR is the adjusted *p*-value, *p* is the original *p*-value of the Spearman correlation, N is the number of ASV pairs, and Rank is the rank of the corresponding original *p*-value among all *p*-values (Benjamini and Hochberg, 1995).

Because many other factors (e.g., diet, crowding effects, etc.) may also affect the co-occurrence index and linear regression is not good at multivariable fitting, we further analyzed the association of the co-occurrence index with all potential variables using the generalized linear model (GLM) implemented in R (R Core Team, 2018) version 4.2.0 with the “glm” function. To avoid the collinearity effect, we first performed a pairwise correlation analysis (Spearman correlation) of those variables, including genetic diversity, Shannon index, Simpson index, chao1, crowding level (average space occupied by an individual), and the number of cohabitating voles (N). We retained only one variable in the GLM analysis for variables with correlation coefficients greater than 0.5. The Simpson index, crowding level, and chao1 were significantly correlated with the Shannon index (Supplementary Figure 1), so we retained only the Shannon index from these variables for the GLM analysis. The initial candidate GLM model included genetic diversity, Shannon index, number cohabitating of voles in the experimental space (N), diet type (categorical variables: grass or chow), and contact type (categorical variables: yes or no). The initial model formulas for the co-occurrence index were as follows:

co-occurrence⁢index⁢G⁢D+d⁢i⁢e⁢t+s⁢h⁢a⁢n⁢n⁢o⁢n+c⁢o⁢n⁢t⁢a⁢c⁢t+N

Here, the co-occurrence index was represented by the Spearman’s correlation coefficient between pairwise ASVs, GD represented the genetic distance between each ASV pair, and diet represented the diet type (i.e., grass or rabbit chow) of the Brandt’s voles in each treatment. The Shannon index represents Shannon diversity (alpha diversity). Contact type indicates whether physical contact between the voles was allowed (non-physical contact or having physical contact) by having or not having barriers in the experimental space. “Non-physical contact” means that cohabitating voles were isolated by barriers that voles can see each other or feel each other through smell, but cannot have physical contact; while “having physical contact” means cohabitating voles can move freely in the experimental space. *N* represents the number of Brandt’s voles in each treatment group. In order to align the scales of the independent and dependent variables and thus reduce potential bias, all variables were normalized using Min-Max method except for the categorical variables of diet and physical contact. The Gaussian family function was used.

We performed automated model selection based on information theory (Burnham and Anderson, 2010) to quantify the relative importance of predictors for co-occurrence index. We ranked the GLM models based on AIC (Akaike’s information criterion) and chose the model with the lowest AIC value among all models as the final model (Supplementary Table 1). The model selection analyses were implemented in R (R Core Team, 2018) version 4.2.0 using the “MuMIn” package. Because factors generated by pairwise comparisons (e.g., co-occurrence index) may have correlated data structure, we randomly selected samples of 1% of the total sample size for re-conducting the GLM analysis. We performed this 10 times and the results were very similar (Supplementary Table 2), indicating that our model results are robust.

Co-occurrence index and genetic distance were calculated by using custom codes in Python^2^ version 3.9.7. The co-occurrence network analysis was performed with the use of CoNet version 1.1.1. Regression analysis was performed with the use of R version 4.2.0.

## Results

### Community composition of the gut microbiota

**FIGURE 1:**
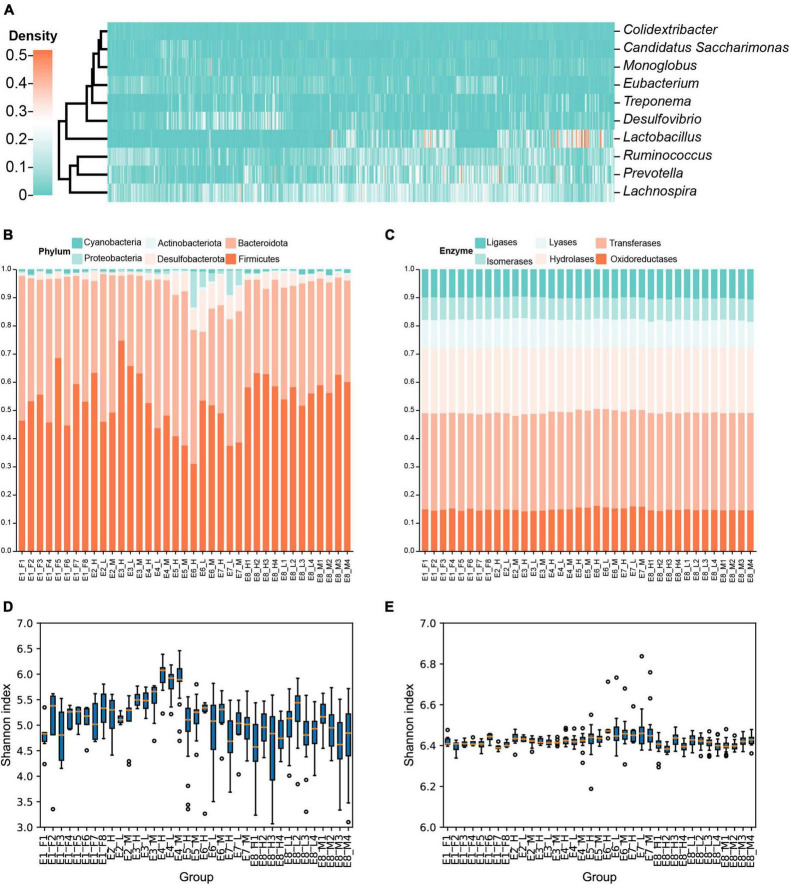
Community composition and diversity of the gut microbiota in Brandt’s vole. **(A)** Abundance of each genus of gut microbiota in each individual of Brandt’s vole. Only genera with an average abundance above 1% were shown. **(B)** Variation in phylum composition of gut microbiota in Brandt’s voles among different treatment groups. Different colors indicate different phyla. **(C)** Function composition of gut microbiota in Brandt’s voles among different treatment groups. Different colors indicate different enzyme classes (level 1 classification). **(D)** Shannon diversity of microbiota of Brandt’s voles in each treatment. **(E)** Functional diversity of microbiota of Brandt’s voles in each treatment. For Simpson diversity, see Supplementary Figure 3.

In total, 4,566 amplicon sequence variants (ASVs) belonging to 116 genera were identified. Three genera (*Desulfovibrio*, *Monoglobus*, and Lachnospiraceae NK4A136 group) were present in all voles, and 32 genera were observed in more than 90% of voles (for details, see Supplementary Data 1). The relative abundances of different genera (e.g., *Desulfovibrio* and *Monoglobus*) varied greatly among the voles (Figure 1A and Supplementary Data 2).

The composition of the gut microbiota varied greatly among different treatments, whereas the functional composition was relatively stable (Figures 1B, C and Supplementary Table 3). Bacteroidota and Firmicutes were the two most abundant phyla in the gut, accounting for the majority of the gut microbiota (Figure 1B); however, their proportions varied greatly among the different treatment groups. For example, the proportion of Firmicutes was more than 74.7% in the E3_H treatment, while the proportion of Bacteroidota was higher (47.5 vs. 31.0%) in the E6_H treatment (Figure 1B). In contrast to the taxon composition, the overall functional composition (Figure 1B) and diversity (Figures 1D, E and Supplementary Data 1) of the gut bacteria showed little variation across the treatment groups.

### Co-occurrence network

**FIGURE 2:**
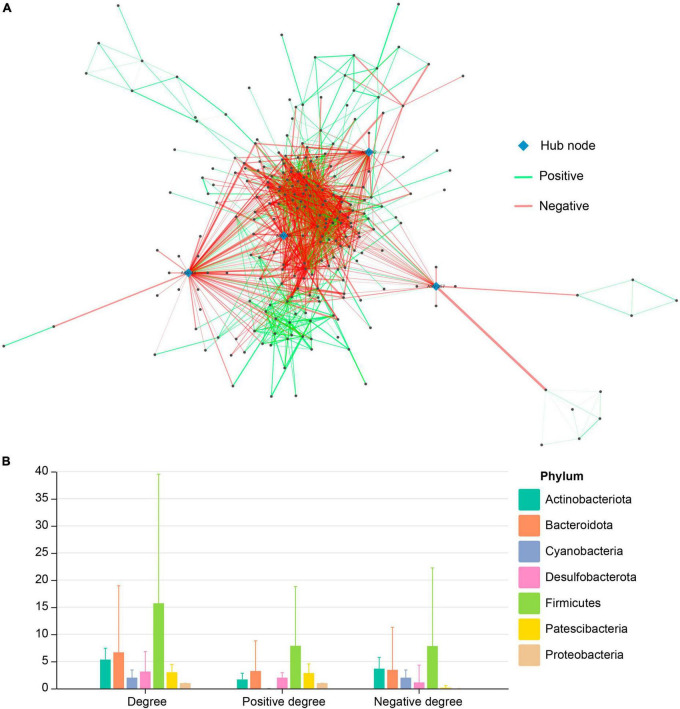
**(A)** Co-occurrence network of pooled ASVs of the gut microbiota in Brandt’s voles under different treatments. **(B)** Distribution of degree, positive degree (i.e., the Spearman correlation coefficient is positive), and negative degree (i.e., the Spearman correlation coefficient is negative) of network nodes in each phylum.

For simplification, we presented only the co-occurrence network of pooled data of gut microbiota from the three studies with significant Spearman’s correlation coefficients (after FDR adjustment). In total, 244 nodes (i.e., ASVs) had significant co-occurrence or co-exclusion relationships with other nodes, resulting in a total of 1,921 edges (i.e., ASV pairs having significant correlation coefficients), of which 920 edges were co-occurrence, and 1,001 were co-exclusion. Among these 244 nodes, four hub nodes were identified (ASV-32, ASV-44, ASV-58, ASV-257), all of which originated from Firmicutes (Figure 2A). Nodes from Firmicutes have the largest average degree, positive degree, and negative degree (Figure 2B). The effects of node degree of other phylum are usually unbalanced, e.g., node degrees from Cyanobacteria are mainly negative, whereas node degrees from Patescibacteria are mainly positive (Figure 2B).

### Factors associated with the co-occurrence index (Spearman correlation coefficient)

**FIGURE 3:**
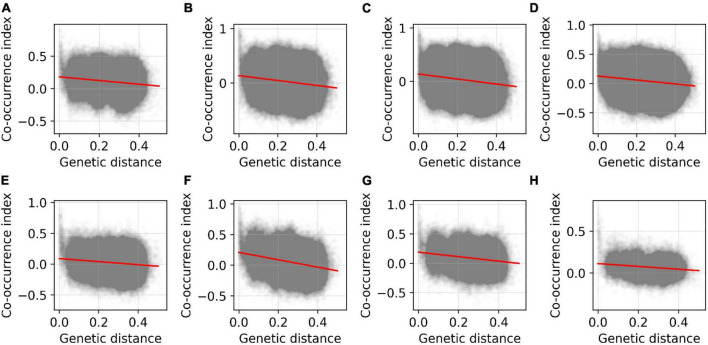
Relationship between co-occurrence index and genetic distance between ASVs of gut microbiota in Brandt’s voles in the eight experiments. The co-occurrence index represents Spearman correlation coefficient, and the scatter points indicate the pooled ASV pairs for each experiment. The red line indicates the linear fitting line. The coefficients of the regression are shown in Supplementary Table 4. **(A–H)** indicate Experiments 1–8 (see “Materials and methods” and Table 1).

Linear regression analysis indicated that the co-occurrence index between ASVs was significantly negatively associated with genetic distance (reversal of genetic relatedness) in all experiments (Figure 3 and Supplementary Table 4) and treatment groups (Supplementary Figure 2 and Supplementary Table 5). The GLM results reconfirmed the negative association between the co-occurrence index and genetic distance after excluding other confounding factors (Table 2), suggesting that more closely related ASVs tend to co-occur in a host.

**TABLE 2:** Coefficients in the GLM analysis of the species co-occurrence index (Spearman correlation).

Dependent	Independent	Estimates	Std error	*t* value	*p*-value
**Co-occurrence index**	Intercept	0.5450	0.0002	2526.9	< 0.001
	GD	−0.0643	0.0003	−228.5	< 0.001
	Shannon index	0.0248	0.0003	92.8	< 0.001
	N	−0.0209	0.0004	−59.3	< 0.001
	Diet: grass	0.0419	0.0001	328.3	< 0.001
	Physical contact: yes	−0.0174	0.0001	−118.3	< 0.001

GD, genetic distance; N, number of cohabitating voles per replicate (experimental space) in each treatment group.

According to the GLM results (Table 2), the number of cohabitating voles in the experimental space was negatively correlated with the co-occurrence index, whereas the alpha diversity of the gut microbiota was positively correlated with the co-occurrence index, suggesting that high diversity may promote ASV co-occurrence. The co-occurrence index was higher in the grass diet treatment group than in the non-grass diet (i.e., rabbit chow) group. The co-occurrence index was higher in the treatment groups that voles cannot contact each other in the experimental space.

## Discussion

Currently, factors and mechanisms that determine species co-existence of gut microbiota have not been elucidated. In this study, using data on the gut microbiota of Brandt’s voles from 37 treatments of eight experiments, we found the species co-occurrence index of gut microbiota in Brandt’s voles was positively associated with the genetic relatedness (reversal of genetic distance) between paired amplicon sequence variants (ASVs), Shannon diversity index, grass diets (natural foods vs. rabbit chow) but negatively associated with the number of cohabitating voles in the experimental space, where a larger number of cohabitating voles represented more crowding stress due to space shortage and more odor or sound stress. Our study demonstrated that high diversity, high genetic relatedness, and favorable environments would benefit species co-occurrence of gut microbiota. Thus, our results provide novel insights into key factors and mechanisms in shaping community assemblies.

### Genetic distance and species co-occurrence

The gut microbial community is a type of commensal community (Artis, 2008), and the co-existence of different kinds of microorganisms is essential for maintaining community stability and function. In ecology, two major hypotheses are used to explain species co-existence or exclusion: the competitive exclusion principle (CEP; similar to niche theory), and the environmental filtering theory (EFT). The CEP emphasizes the effects of interspecific interactions, whereas the EFT focuses on the dependence or response of species to similar environments and resources. Genetic relatedness is often used to test the EFT and EFT has been supported by many studies; for example, Yan et al. (2016) found that the species co-occurrence of mammals, birds, reptiles, and amphibians in China was negatively associated with the genetic distance between species (Yan et al., 2016). Wang et al. (2022) also found that species (Lepidoptera) co-occurrence is negatively associated with their phylogenetic relatedness (i.e., genetic distance) (Wang et al., 2022). These two theories are not exclusive to each other but may be complementary at different scales or taxa, for example, at a small spatial scale. Wang et al. (2022) found that Lepidoptera co-existence is lower at smaller spatial scales, providing evidence in support of the CEP on a small spatial scale, where species may compete more seriously with each other. To the best of our knowledge, no studies have been conducted to test the EFT using gut microbiota. In this study, we found that genetic distance is negatively associated with the co-occurrence index in the gut microbes of Brandt’s voles, providing new evidence in support of the EFT. Bacteria with close genetic relatedness may tend to have similar functions and require similar resources or environments; therefore, they tend to have a similar response to environmental changes (such as diet and stress), as predicted by the EFT. Notably, genetic relatedness does not always represent trait similarity, so further studies on the relationship between function similarity and genetic distance are necessary. Competition and colonization trade-off has been suggested to affect community assembly (Bin et al., 2019) because competitive ability and colonization capacity may lead to ecological niche differences. Unfortunately, we have no data on the colonization ability of gut microbes, which requires further study. It should be pointed out that the co-occurrence index of species may cover various species interactions (e.g., mutualism, predation) and may not be used to test the CEP theories that emphasize species sharing similar resources.

### Diversity and species co-occurrence

Many studies have suggested that diversity can promote the stability of communities (Girvan et al., 2005; Proulx et al., 2010; Cadotte et al., 2012), ecosystem reliability (Naeem and Li, 1997), and the stability of ecosystem functions at various ecological scales (Oliver et al., 2015), which may also benefit species co-occurrence. A larger number of species in the trophic network facilitates the provision of more food sources, thereby maintaining the stability of the network (Yang et al., 2022). One species may likely depend heavily on several resource species that are complementary or competitive with each other; thus, more diversified resource species may facilitate the coexistence of consumer species (Naeem and Li, 1997; Yang et al., 2022). A previous study demonstrated that the gut bacterial composition of migratory birds varied greatly among individuals, but their microbiome metabolism and functions were similar (Cao et al., 2020). Similarly, we found that the composition of gut microbiota varied considerably under different treatments, but the functional composition was more stable (Figure 1), suggesting that the functions of the diversified bacteria were complementary to each other; thus, high diversity helps maintain the coexistence of gut microbes.

In this study, we found that the diversity of gut microbiota in Brandt’s voles was positively associated with species co-occurrence index (Table 2). Therefore, we argue that communities with high diversity have more ecological niches to offer, and can therefore favor the coexistence of more species.

### Environmental factors and species co-occurrence

As discussed above, high diversity would produce more positive relations between species, increasing biomass or productivity, thus facilitating species co-occurrence. However, unfavorable environments often reduce diversity (Li et al., 2019, 2021a; Liu et al., 2020, 2022), thus, harm species co-occurrence. The positive Spearman correlation either indicates mutualism or the same response to a favorable environmental variable (e.g., food type, stress condition), while the negative Spearman correlation either indicates competition or predation, or the same response to unfavorable environmental variables. In this study, we found that the species co-occurrence index of gut microbiota in Brandt’s voles was positively associated with grass diets (i.e., natural food) and no-physical contact within the experimental space, but negatively associated with the number of cohabitating voles in the experimental space. A larger number of cohabitating voles in one experimental space often represents more crowding stress due to space shortage, stronger odor, or auditory stress, while no barrier between voles would increase social stress due to direct fighting (Liu et al., 2022), which may result in dysfunction of gut microbiota and a harmful impact on the species co-occurrence of ASVs. In this study, each experiment consisted of several treatments, while each treatment contained several replicates. The voles in each replicate were co-housed (with or without physical contact between individuals) and experienced the same treatment pressure (e.g., density).

Grass diets are natural for Brandt’s voles and may be more diversified in nutrients (e.g., fiber) compared to rabbit chow, thus favoring the co-occurrence of ASVs. Furthermore, natural foraging allows for a more diverse diet, which may create a more diverse nutritional environment in the gut that is conducive to a broader range of microbial taxa (Heiman and Greenway, 2016). Nutrients, such as fiber, fat, and protein, have significant effects on the composition and function of intestinal microorganisms (Wu et al., 2022). Low-fiber diets can trigger a significant depletion of gut microbial diversity and beneficial metabolites, disrupting the composition of gut microbiota (Riva et al., 2019). Dietary supplementation with functional amino acids, including tryptophan, glutamine, methionine, and branched-chain amino acids, significantly optimizes the structure and function of the microbial community and establishes a new balance of host-microbiota interactions (Ma and Ma, 2019). Li et al. (2019) found that voles in livestock grazing enclosures exhibited significantly lower alpha diversity, and that the microbiota from voles in grazed enclosures had a smaller and more simplified co-occurrence network with a relatively higher percentage of positive interactions. Li et al. (2021a) found that the alpha diversity of gut microbiota increased linearly with dietary species richness, whereas dietary species richness affected the composition, function, and community assembly of the gut microbiota of Brandt’s voles in a nonlinear manner. These results indicate that favorable and more diversified natural plants are beneficial to the growth of Brandt’s voles through their gut microbiota.

### Implications of this study

Dysbiosis and diseases may be linked to the loss of diversity in host gut microbes (Kriss et al., 2018). Our study revealed that a high diversity of gut microbiota could increase the species co-occurrence of the gut microbiota. Thus, biodiversity preservation of gut microbes is essential for maintaining the host’s health. We found that unfavorable conditions (e.g., poor-quality food and high-stress living conditions) decreased species co-occurrence of gut microbes, suggesting that they may be important intrinsic (density dependency effect) or external (food) factors in regulating the composition of gut microbiota.

Notably, technical biases, stochastic responses of the gut microbiota to the altered gut environment, and intrinsic properties of the gut microbiota may lead to different results. Thus, more studies are needed to investigate the underlying mechanism in regulating species coexistence of gut microbiota (Costea et al., 2017; Zaneveld et al., 2017; Poyet et al., 2019).

## Data availability statement

The original contributions presented in this study are included in this article/Supplementary material, further inquiries can be directed to the corresponding author.

## Ethics statement

The study was conducted in accordance with the guidelines by the Animal Care and Use Committee of the Institute of Zoology of Chinese Academy of Sciences.

## Author contributions

CC: Conceptualization, Data curation, Software, Visualization, Writing – original draft, Writing – review & editing. GL: Data curation, Writing – review & editing. XY: Writing – review & editing. JZ: Writing – review & editing, Data curation. JL: Data curation, Writing – review & editing. AZ: Writing – review & editing. ZZ: Conceptualization, Funding acquisition, Writing – original draft, Writing – review & editing.

## Footnotes

1
https://www.arb-silva.de/

2
https://www.python.org/
