# Supplementary material for: High diversity, close genetic relatedness, and favorable living conditions benefit species co-occurrence of gut microbiota in Brandt’s vole
Source: Front Microbiol. 2024 Feb 7;15:1337402. doi: 10.3389/fmicb.2024.1337402 (PMC10879610; doi:10.3389/fmicb.2024.1337402)
Supplement: Supplementary file 1 [file Data_Sheet_1.docx]

***Supplementary Material***

1. **Experiments information**
   1. **Experiment 1 (Study 1): Diet manipulation experiment.** This experiment aimed to test the effects of dietary species richness (DSR) in artificial food diets containing plant species commonly consumed by Brandt’s voles (*L. chinensis, M. sativa, A. polyrhizum, C. ammannii, S. krylovii, C. squarrosa, C. aristatum,* and *P. dentosa*). The artificial food was prepared using different combinations of the eight plant species, and the experiment consisted of eight diet treatment groups. We added more species to the diet in the order of plants with high to low palatability, and there were five to six voles (replicates) in each treatment group (Table S1). Voles were raised individually with artificial food and tap water for four weeks before they were sacrificed, and their feces were collected at the end of the experiment. For more details, see (Li et al., 2021).
   2. **Experiment 2 (Study 2): Crowding experiment without physical contact**. This experiment aimed to test crowding effects without physical contact under laboratory conditions. Each vole was housed in a single cage (L × W × H = 24 × 11.5 × 10 cm), and crowding effect was simulated by placing several cages together without physical contact. A total of 45 male voles were housed individually in 45 cages and divided into three treatment groups: a low-crowding (LC), a medium-crowding (MC), and a high-crowding (HC) groups. The LC group consisted of two cages placed close to each other (four replicates), with one vole in each cage, and each vole had only one neighbor. The MC group consisted of four cages placed close to each other (three replicates), with one vole in each cage and three neighbors per vole. The HC group consisted of 25 cages placed close to each other, with one vole in each cage. The experiment lasted for four weeks, and rabbit chow and tap water were freely supplied to the voles. For more details, see (Liu et al., 2020).
   3. **Experiment 3 (Study 2): Crowding experiment with physical contact**. The experiment aimed to test crowding effects with physical contact, in which voles in the same cage had free access to each other. A total of 56 male voles were divided into three treatment groups: low-density (LD), medium-density (MD), and high-density (HD) groups. The LD group consisted of two voles in one plastic box (48 × 25 × 20 cm) with eight replicates. The MD group consisted of four voles in a plastic box with four replicates. The HD group consisted of eight voles in a plastic box with three replicates. Rabbit chow and tap water were supplied to voles for free feeding. The test lasted for four weeks, and feces were collected from the colon at the end of the experiment to sequence the gut microbes. For more details, see (Liu et al., 2020) and Table S1.
   4. **Experiment 4 (Study 3): Crowding experiment** **with physical contact and space shortage.** This experiment aimed to test the crowding effects of an increasing number of voles with physical contact and space shortages in the laboratory. The experiment included three treatment groups that contained one, two, and four paired voles (each pair had one male and one female), and all groups were housed in plastic boxes of the same size (26 × 15 × 14 cm). The LC group consisted of one pair of voles with 12 replicates. The MC group consisted of two pairs of voles with six replicates. The HC group consisted of four pairs of voles with three replicates. Rabbit chow and tap water were supplied to the voles *ad libitum*. The experiment lasted for four weeks, and colonic feces were collected at the end of the experiment. For details, see (Liu et al., 2022) and Table S1.
   5. **Experiment 5 (Study 3): Crowding experiment without physical contact but with space shortage.** This experiment aimed to test the crowding effects of an increasing number of voles without physical contact but with a space shortage in the laboratory. The experiment consisted of three treatment groups containing one, two, and four paired voles (each pair had one male and one female). The movement of each pair of voles was restricted to a smaller space using a plastic plate in the plastic box (26 × 15 × 14 cm). The LC group consisted of one pair of voles with 12 replicates. The MC group consisted of two pairs of voles with six replicates. The HC group consisted of four pairs of voles with three replicates. Rabbit chow and water were supplied to the voles *ad libitum*. The experiment lasted four weeks, and colonic feces were collected at the end of the experiment. For details, (Liu et al., 2022) and Table S1.
   6. **Experiment 6 (Study 3): Crowding experiment with physical contact and space shortage.** This experiment aimed to test the crowding effects of an increasing number of voles with physical contact and space shortages in the laboratory. The experiment consisted of three treatment groups containing one, two, and four paired voles (each pair had one male and one female). The LC group consisted of one pair of voles in a small plastic box (26 × 15 × 14 cm) with 12 replicates. The MC group consisted of two pairs of voles in a medium-sized plastic box (26 × 30 × 14 cm) with six replicates. The HC group consisted of four pairs of voles in a large plastic box (52 × 30 × 14 cm) with three replicates. Rabbit chow and tap water were supplied to the voles *ad libitum*. The experiment lasted four weeks, and the colonic feces were collected at the end. For details, see (Liu et al., 2022) and Table S1.
   7. **Experiment 7 (Study 3): Crowding experiment without physical contact or space shortage.** This experiment aimed to test the crowding effects of an increasing number of voles without physical contact or space shortages in the laboratory. The experiment consisted of three treatment groups that contained one, two, and four paired voles (each pair had one male and one female). The movement of each pair of voles was restricted to a smaller space using a plastic plate in the plastic box. The LC group consisted of one pair of voles in a small plastic box (26 × 15 × 14 cm) with 12 replicates. The MC group consisted of two pairs of voles in a medium-sized plastic box (26 × 30 × 14 cm) with six replicates. The HC group consisted of four pairs of voles in a large plastic box (52 × 30 × 14 cm) with three replicates. Rabbit chow and water were supplied to the voles *ad libitum*. The experiment lasted four weeks, and the colonic feces were collected at the end. For details, see (Liu et al., 2022) and Table S1.
   8. **Experiment 8 (Study 3): Crowding experiment with physical contact and space shortage.** The experiment aimed to test the crowding effects of space shortage (simulated by LD, MD, and HD groups in the same-sized enclosures) with physical contact (voles moving freely in each enclosure) in semi-natural enclosures. The enclosure trial was conducted at the Inner Mongolia Research Station of Animal Ecology (44°119 N, 116°279 E) in Xilinhot, Inner Mongolia, China, which is located in a typical steppe grassland. Twelve enclosures (60 × 80 m) were used to conduct the experiment. The enclosures were constructed to prevent the immigration of voles across the enclosures and predation by birds or other predators. The enclosures were divided into three density groups (four replicates per group). We released six pairs, 12 pairs, and 24 pairs of adult male and female founder voles into the LD, MD, and HD enclosures, respectively, in early spring; the voles bred naturally in the enclosures during the breeding season. The experiment lasted from April 1 to October 1, 2019, and the feces of each vole were collected at the end of the experiment. Voles were free to feed on natural plants in the enclosure. We also provided 150 g of rabbit chow to each vole family once a week to ensure that each individual had a sufficient food supply. For details, see (Liu et al., 2022) and Table S1.

**2 Supplementary Figures and Tables**

**Supplementary Fig. S1**


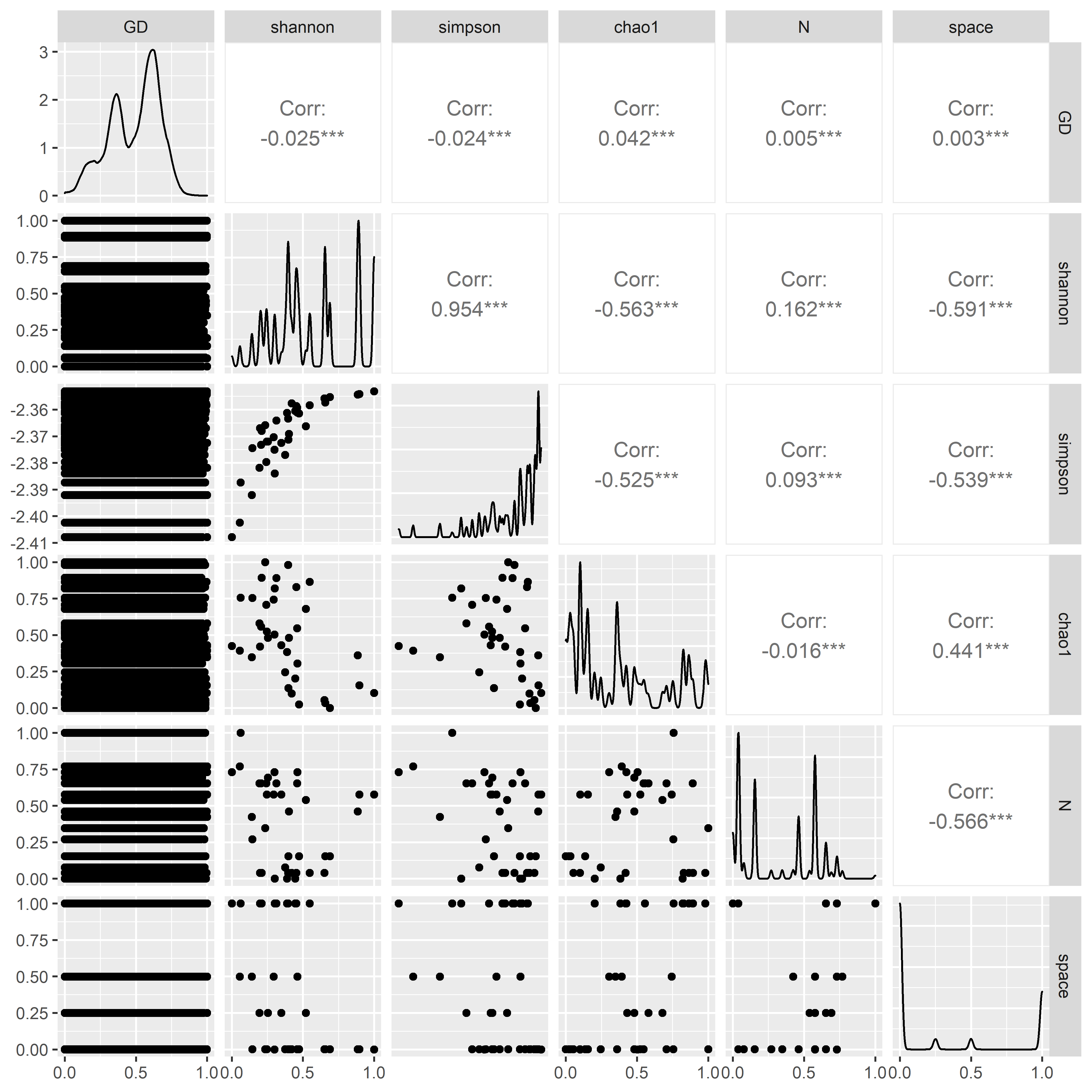


**Fig. S1. Spearman correlation between variables used in GLM analysis for detecting potential collinearity.**

**Supplementary Fig. S2**


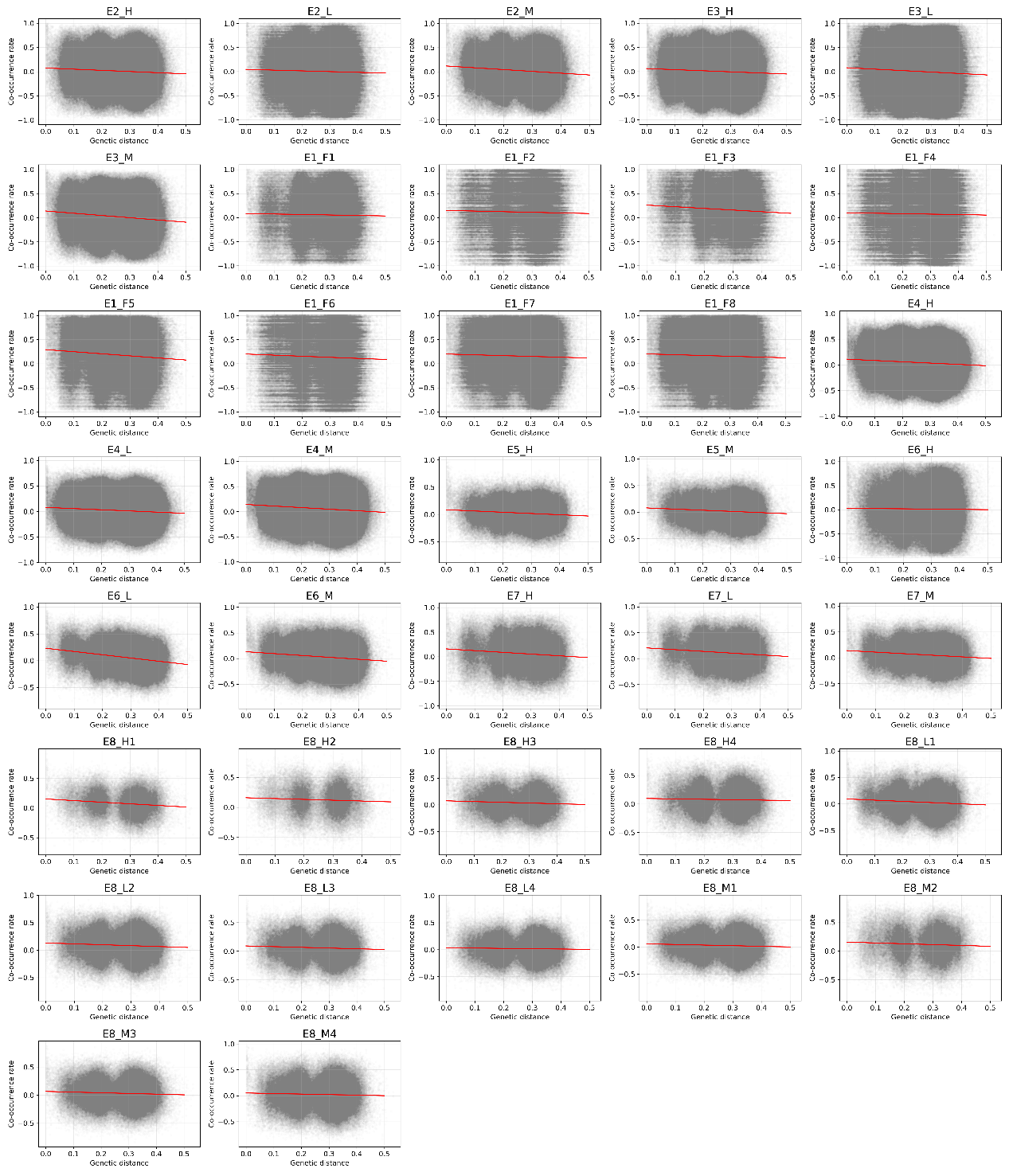


**Fig. S2. Relationship between co-occurrence index and genetic distance** **between ASV pairs.** The x-axis indicates the genetic distance, the y-axis indicates the spearman correlation coefficient, and the scatter indicates the ASV pairs. The red line indicates the linearly fitted line. The coefficients of the regression are shown in Table S3.

**Supplementary Fig. S3**


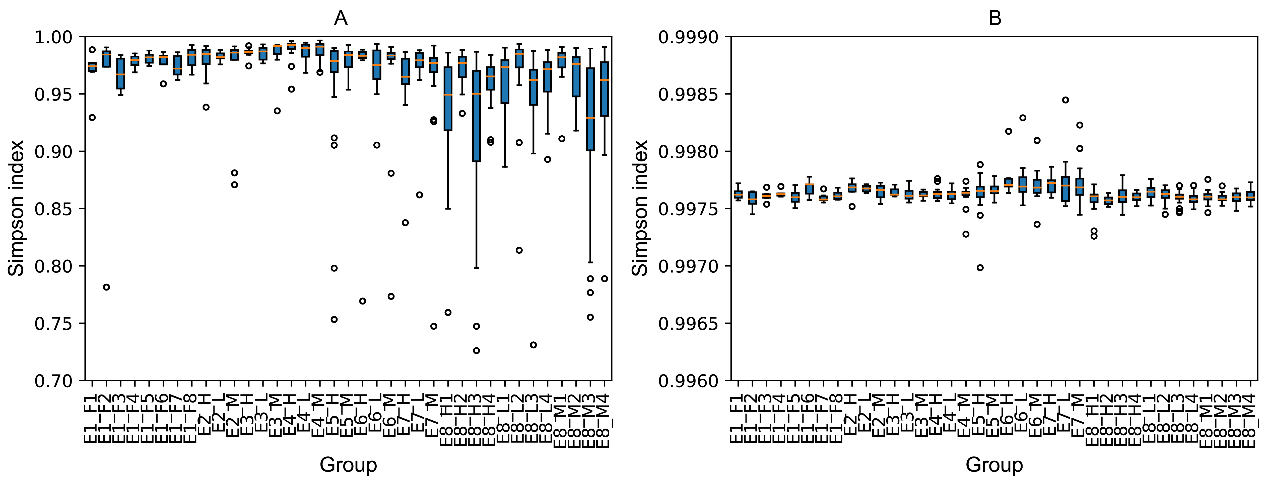


**Fig. S3**. **ASV Simpson diversity (A) and functional diversity (B) of Brandt’s voles in different treatments**.

**Reference**

Li, G., Shi, C., Song, Y., Chu, H., & Zhang, Z. (2021). The Role Transition of Dietary Species Richness in Modulating the Gut Microbial Assembly and Postweaning Performance of a Generalist Herbivore. *MSystems*, *6*(6), e00979-21. https://doi.org/10.1128/mSystems.00979-21

Liu, J., Huang, S., Li, G., Zhao, J., Lu, W., & Zhang, Z. (2020). High housing density increases stress hormone- or disease-associated fecal microbiota in male Brandt’s voles (Lasiopodomys brandtii). *Hormones and Behavior*, *126*, 104838. https://doi.org/10.1016/j.yhbeh.2020.104838

Liu, J., Huang, S., Zhang, X., Li, G., Batsuren, E., Lu, W., Xu, X., He, C., Song, Y., & Zhang, Z. (2022). Gut microbiota reflect the crowding stress of space shortage, physical and non-physical contact in Brandt’s voles (Lasiopodomys brandtii). *Microbiological Research*, *255*, 126928. https://doi.org/10.1016/j.micres.2021.126928

**Supplementary Table S1. Model selection results of models with combinations of variables in the global model.** Y represents the dependent variables in the GLM analysis. AIC represents the Akaike’s information criterion. Each row represents a model, and “+” indicates that the model of that row contains the corresponding variable, while “NA” indicates that it does not contain the corresponding variable.

| Y | Physical contact | Diet | GD | N | Shannon index | df | AIC |
| --- | --- | --- | --- | --- | --- | --- | --- |
| Spearman | + | + | + | + | + | 7 | -7920931.031 |
| Spearman | + | + | + | + | NA | 6 | -7918536.46 |
| Spearman | NA | + | + | + | + | 6 | -7915819.318 |
| Spearman | NA | + | + | + | NA | 5 | -7910019.483 |
| Spearman | + | + | + | NA | + | 6 | -7909717.274 |
| Spearman | NA | + | + | NA | + | 5 | -7907998.974 |
| Spearman | + | + | + | NA | NA | 5 | -7907190.103 |
| Spearman | NA | + | + | NA | NA | 4 | -7903358.332 |
| Spearman | + | + | NA | + | + | 6 | -7870567.316 |
| Spearman | + | + | NA | + | NA | 5 | -7867782.827 |

**Supplementary Table S2. Coefficients in the GLM analysis of the species co-occurrence index (Spearman correlation) using resampled data (1% of total samples).** GD: genetic distance; N: number of cohabitating voles per replicate (experimental space) in each treatment group.

| Independent | Estimates | Std Error | *t* value |
| --- | --- | --- | --- |
| Intercept | 0.5458 | 0.0022 | 252.4 |
| GD | -0.0650 | 0.0028 | -23.1 |
| Diet: grass | 0.0399 | 0.0013 | 31.2 |
| Contact: yes | -0.0168 | 0.0015 | -11.4 |
| Shannon | 0.0248 | 0.0027 | 9.3 |
| N | -0.0197 | 0.0035 | -5.6 |
| Intercept | 0.5443 | 0.0022 | 252.9 |
| GD | -0.0655 | 0.0028 | -23.4 |
| Diet: grass | 0.0442 | 0.0013 | 34.7 |
| Contact: yes | -0.0161 | 0.0015 | -10.9 |
| Shannon | 0.0261 | 0.0027 | 9.8 |
| N | -0.0246 | 0.0035 | -7.0 |
| Intercept | 0.5457 | 0.0022 | 252.8 |
| GD | -0.0625 | 0.0028 | -22.2 |
| Diet: grass | 0.0407 | 0.0013 | 31.8 |
| Contact: yes | -0.0159 | 0.0015 | -10.8 |
| Shannon | 0.0226 | 0.0027 | 8.5 |
| N | -0.0244 | 0.0035 | -7.0 |
| Intercept | 0.5446 | 0.0022 | 251.5 |
| GD | -0.0611 | 0.0028 | -21.7 |
| Diet: grass | 0.0405 | 0.0013 | 31.6 |
| Contact: yes | -0.0202 | 0.0015 | -13.6 |
| Shannon | 0.0260 | 0.0027 | 9.7 |
| N | -0.0188 | 0.0035 | -5.3 |
| Intercept | 0.5436 | 0.0022 | 252.2 |
| GD | -0.0649 | 0.0028 | -23.1 |
| Diet: grass | 0.0437 | 0.0013 | 34.2 |
| Contact: yes | -0.0184 | 0.0015 | -12.5 |
| Shannon | 0.0267 | 0.0027 | 10.1 |
| N | -0.0187 | 0.0035 | -5.3 |
| Intercept | 0.5479 | 0.0022 | 253.7 |
| GD | -0.0699 | 0.0028 | -24.8 |
| Diet: grass | 0.0419 | 0.0013 | 32.8 |
| Contact: yes | -0.0178 | 0.0015 | -12.1 |
| Shannon | 0.0258 | 0.0027 | 9.6 |
| N | -0.0204 | 0.0035 | -5.8 |
| Intercept | 0.5411 | 0.0022 | 250.3 |
| GD | -0.0587 | 0.0028 | -20.8 |
| Diet: grass | 0.0436 | 0.0013 | 34.1 |
| Contact: yes | -0.0145 | 0.0015 | -9.8 |
| Shannon | 0.0225 | 0.0027 | 8.4 |
| N | -0.0235 | 0.0035 | -6.7 |
| Intercept | 0.5431 | 0.0022 | 251.1 |
| GD | -0.0605 | 0.0028 | -21.4 |
| Diet: grass | 0.0419 | 0.0013 | 32.9 |
| Contact: yes | -0.0189 | 0.0015 | -12.9 |
| Shannon | 0.0266 | 0.0027 | 9.9 |
| N | -0.0211 | 0.0035 | -6.0 |
| Intercept | 0.5443 | 0.0022 | 252.5 |
| GD | -0.0646 | 0.0028 | -23.0 |
| Diet: grass | 0.0411 | 0.0013 | 32.2 |
| Contact: yes | -0.0171 | 0.0015 | -11.6 |
| Shannon | 0.0243 | 0.0027 | 9.1 |
| N | -0.0129 | 0.0035 | -3.7 |
| (Intercept) | 0.5453 | 0.0022 | 253.3 |
| GD | -0.0652 | 0.0028 | -23.2 |
| Diet: grass | 0.0428 | 0.0013 | 33.4 |
| Contact: yes | -0.0154 | 0.0015 | -10.5 |
| Shannon | 0.0236 | 0.0027 | 8.9 |
| N | -0.0259 | 0.0035 | -7.4 |

**Supplementary Table S3. Abundance variability (*CV*) of different phyla of Brandt’s voles among treatments.**

| Phylum | Average abundance (Proportion) | Standard deviation | *CV* |
| --- | --- | --- | --- |
| Firmicutes | 0.5137 | 0.0950 | 0.1848 |
| Bacteroidota | 0.3858 | 0.0750 | 0.1943 |
| Desulfobacterota | 0.0345 | 0.0331 | 0.9604 |
| Spirochaetota | 0.0192 | 0.0134 | 0.6956 |
| Patescibacteria | 0.0148 | 0.0100 | 0.6742 |
| Proteobacteria | 0.0105 | 0.0196 | 1.8550 |
| Cyanobacteria | 0.0075 | 0.0044 | 0.5892 |
| Verrucomicrobiota | 0.0060 | 0.0087 | 1.4500 |
| Actinobacteriota | 0.0049 | 0.0030 | 0.6159 |
| Campilobacterota | 0.0017 | 0.0016 | 0.9712 |
| Elusimicrobiota | 0.0012 | 0.0014 | 1.1022 |

**Supplementary Table S4. Linear fitted parameters of co-occurrence index (Spearman correlation coefficient) and genetic distances between ASV pairs for different experiments**. *r*, the correlation coefficient between co-occurrence index and genetic distance. SD, standard deviation.

| dataset | slope | intercept | *r* | *p-value* | SD |
| --- | --- | --- | --- | --- | --- |
| Experiment 1 | -0.2837 | 0.1815 | -0.1530 | < 0.001 | 0.0044 |
| Experiment 2 | -0.4506 | 0.1360 | -0.1773 | < 0.001 | 0.0038 |
| Experiment 3 | -0.4693 | 0.1388 | -0.1865 | < 0.001 | 0.0029 |
| Experiment 4 | -0.3364 | 0.1279 | -0.1640 | < 0.001 | 0.0020 |
| Experiment 5 | -0.2495 | 0.0910 | -0.1379 | < 0.001 | 0.0040 |
| Experiment 6 | -0.5999 | 0.2091 | -0.2941 | < 0.001 | 0.0038 |
| Experiment 7 | -0.3827 | 0.1888 | -0.2060 | < 0.001 | 0.0047 |
| Experiment 8 | -0.1681 | 0.1133 | -0.1742 | < 0.001 | 0.0034 |

**Supplementary Table S5. Linear fitted parameters of co-occurrence index (Spearman correlation coefficients) and genetic distances between ASV pairs for different treatment groups.** *r*, the correlation coefficient between co-occurrence index and genetic distance. SD, standard deviation.

| Treatment | Slope | Intercept | *r* | *p-value* | SD |
| --- | --- | --- | --- | --- | --- |
| E1_F1 | -0.0965 | 0.0841 | -0.0200 | < 0.001 | 0.0094 |
| E1_F2 | -0.1277 | 0.1490 | -0.0240 | < 0.001 | 0.0100 |
| E1_F3 | -0.3423 | 0.2601 | -0.0718 | < 0.001 | 0.0095 |
| E1_F4 | -0.0942 | 0.1007 | -0.0174 | < 0.001 | 0.0085 |
| E1_F5 | -0.4309 | 0.2918 | -0.0864 | < 0.001 | 0.0075 |
| E1_F6 | -0.2248 | 0.1994 | -0.0397 | < 0.001 | 0.0098 |
| E1_F7 | -0.1611 | 0.2000 | -0.0337 | < 0.001 | 0.0067 |
| E1_F8 | -0.1590 | 0.2022 | -0.0324 | < 0.001 | 0.0063 |
| E2_H | -0.2421 | 0.0758 | -0.0625 | < 0.001 | 0.0056 |
| E2_L | -0.1525 | 0.0452 | -0.0311 | < 0.001 | 0.0073 |
| E2_M | -0.3712 | 0.1180 | -0.0948 | < 0.001 | 0.0063 |
| E3_H | -0.2250 | 0.0623 | -0.0578 | < 0.001 | 0.0046 |
| E3_L | -0.2989 | 0.0800 | -0.0612 | < 0.001 | 0.0056 |
| E3_M | -0.4638 | 0.1395 | -0.1164 | < 0.001 | 0.0049 |
| E4_H | -0.2539 | 0.1108 | -0.0830 | < 0.001 | 0.0027 |
| E4_L | -0.2315 | 0.0798 | -0.0827 | < 0.001 | 0.0027 |
| E4_M | -0.3148 | 0.1429 | -0.1000 | < 0.001 | 0.0030 |
| E5_H | -0.2292 | 0.0853 | -0.0953 | < 0.001 | 0.0052 |
| E5_M | -0.2163 | 0.0787 | -0.0878 | < 0.001 | 0.0052 |
| E6_H | -0.0587 | 0.0310 | -0.0132 | < 0.001 | 0.0075 |
| E6_L | -0.6075 | 0.2319 | -0.2316 | < 0.001 | 0.0051 |
| E6_M | -0.3684 | 0.1322 | -0.1283 | < 0.001 | 0.0052 |
| E7_H | -0.3515 | 0.1593 | -0.1062 | < 0.001 | 0.0077 |
| E7_L | -0.3326 | 0.2064 | -0.1116 | < 0.001 | 0.0068 |
| E7_M | -0.3043 | 0.1396 | -0.1168 | < 0.001 | 0.0056 |
| E8_H1 | -0.2718 | 0.1535 | -0.1263 | < 0.001 | 0.0081 |
| E8_H2 | -0.1371 | 0.1601 | -0.0547 | < 0.001 | 0.0104 |
| E8_H3 | -0.1366 | 0.0753 | -0.0543 | < 0.001 | 0.0074 |
